# Hematologic dysfunction in cancer: Mechanisms, effects on antitumor immunity, and roles in disease progression

**DOI:** 10.3389/fimmu.2022.1041010

**Published:** 2022-12-06

**Authors:** Viktoria Plackoska, Dania Shaban, Anastasia Nijnik

**Affiliations:** ^1^ Department of Physiology, McGill University, Montreal, QC, Canada; ^2^ McGill University Research Centre on Complex Traits, McGill University, Montreal, QC, Canada

**Keywords:** cancer, hematopoiesis, myelopoiesis, erythropoiesis, immune cell development, hematopoietic stem and progenitor cells, myeloid-derived suppressor cells, tumor associated macrophages

## Abstract

With the major advances in cancer immunology and immunotherapy, it is critical to consider that most immune cells are short-lived and need to be continuously replenished from hematopoietic stem and progenitor cells. Hematologic abnormalities are prevalent in cancer patients, and many ground-breaking studies over the past decade provide insights into their underlying cellular and molecular mechanisms. Such studies demonstrate that the dysfunction of hematopoiesis is more than a side-effect of cancer pathology, but an important systemic feature of cancer disease. Here we review these many advances, covering the cancer-associated phenotypes of hematopoietic stem and progenitor cells, the dysfunction of myelopoiesis and erythropoiesis, the importance of extramedullary hematopoiesis in cancer disease, and the developmental origins of tumor associated macrophages. We address the roles of many secreted mediators, signaling pathways, and transcriptional and epigenetic mechanisms that mediate such hematopoietic dysfunction. Furthermore, we discuss the important contribution of the hematopoietic dysfunction to cancer immunosuppression, the possible avenues for therapeutic intervention, and highlight the unanswered questions and directions for future work. Overall, hematopoietic dysfunction is established as an active component of the cancer disease mechanisms and an important target for therapeutic intervention.

## Introduction

**Figure 1:**
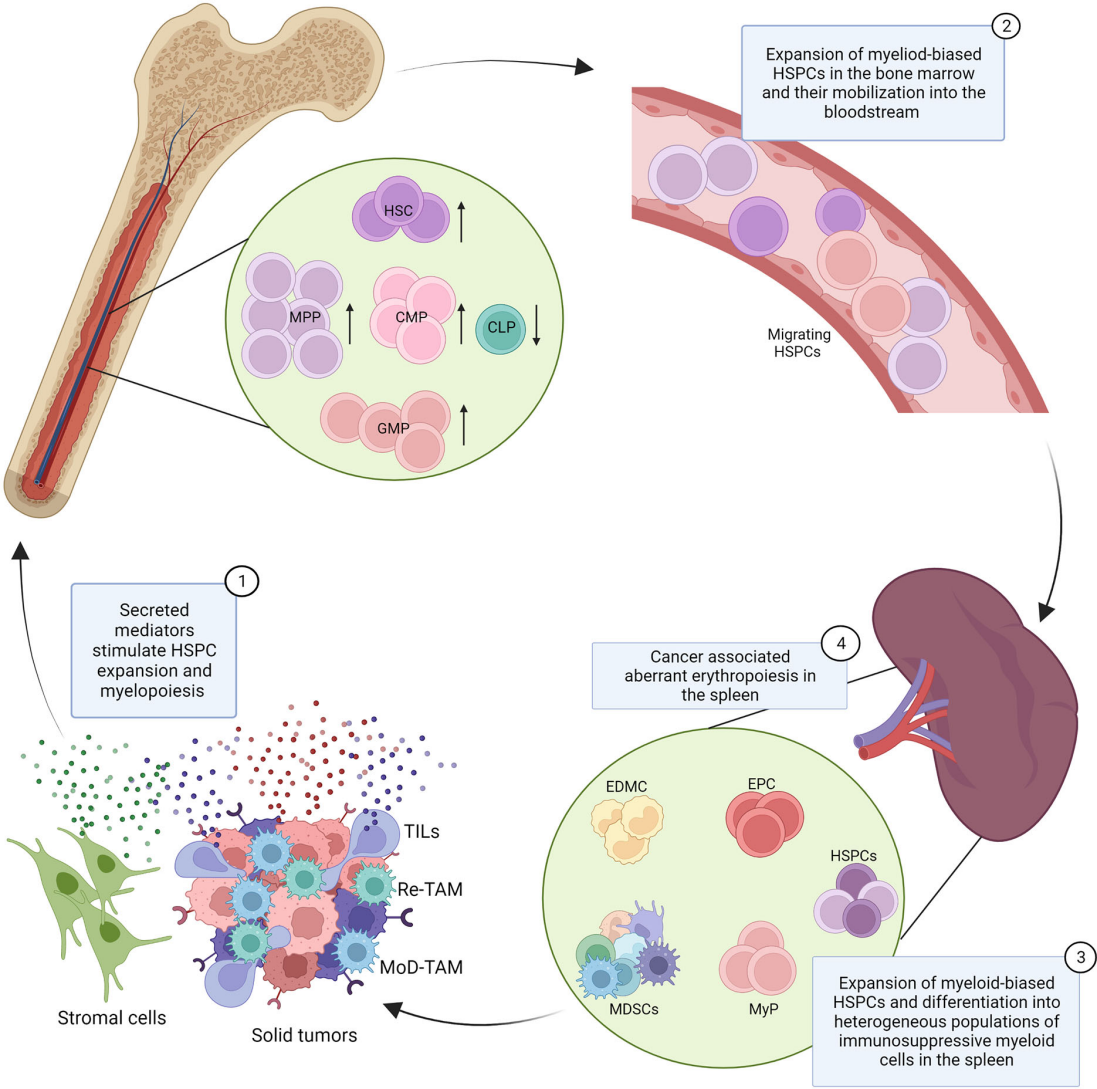
Dysfunction of hematopoiesis in cancer. Secreted mediators produced by cancer cells, tumor infiltrating leukocytes (TILs), and stromal cells (1) promote the expansion of hematopoietic stem cells (HSC), multipotent progenitors (MPPs), common myeloid progenitors (CMPs), and granulocyte monocyte progenitors (GMPs) in the bone marrow and their mobilization into the bloodstream (2). The cancer associated induction of extramedullary hematopoiesis is characterized by the expansion of hematopoietic stem and progenitor cells (HSPCs) and myeloid progenitor cells (MyPs) in the spleen and their local differentiation into heterogeneous populations of myeloid cells with immunosuppressive properties, known as tumor associated neutrophils/monocytes or myeloid-derived suppressor cells (MDSCs) (3). Cancer associated myelopoiesis also contributes to the production of monocyte-derived tumor associated macrophages (MoD-TAMs). Splenic extramedullary erythropoiesis is also reported in cancer, characterized by the expansion of erythroid progenitor cells (EPCs) in the spleen and the production of erythroid differentiated myeloid cells (EDMCs), with potent immunosuppressive properties (4). This model integrates findings from multiple cancer types and experimental systems, and the indicated mechanisms may not be conserved across all cancers.

**Figure 2:**
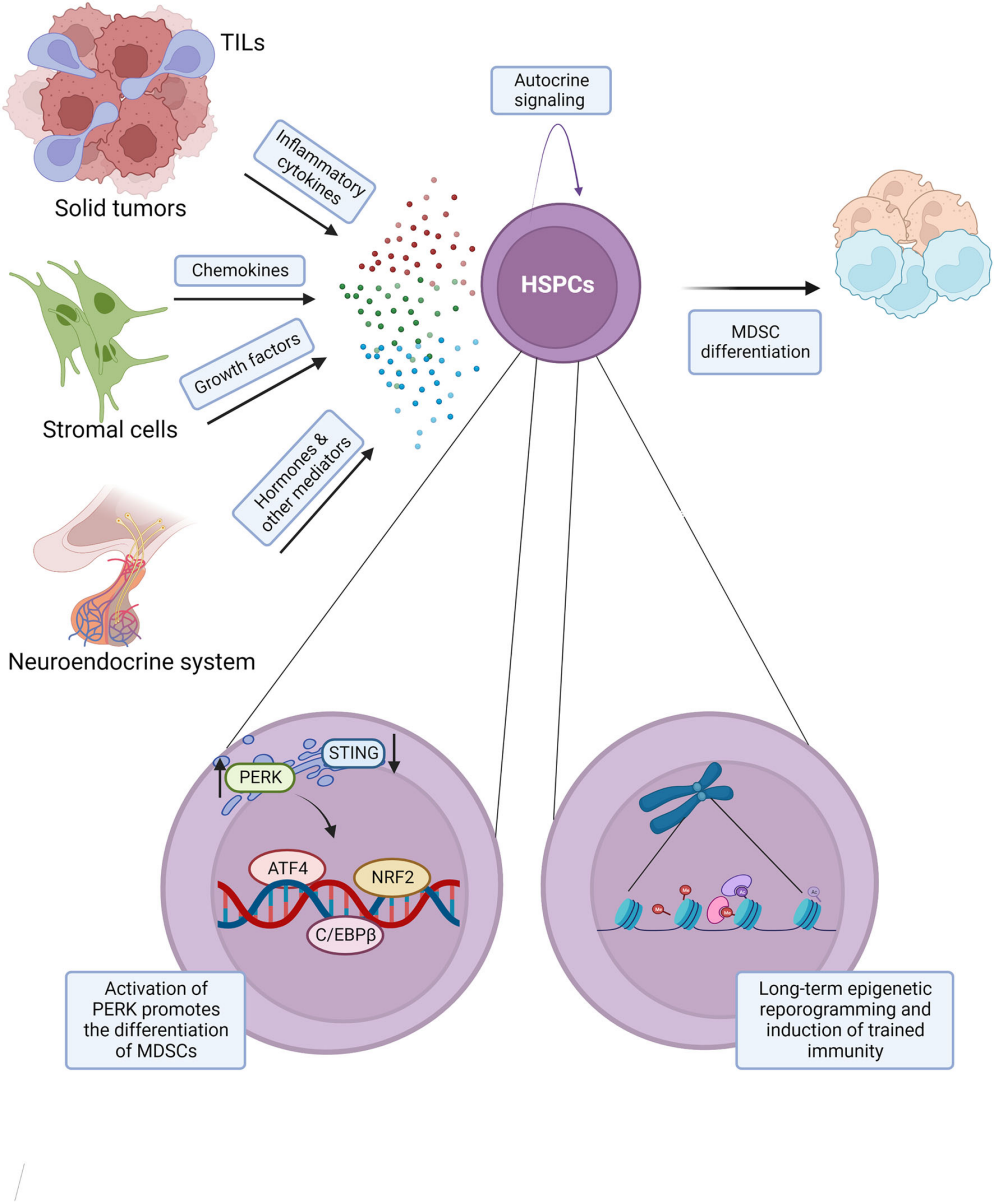
Molecular regulation of cancer associated myelopoiesis. Secreted mediators promoting cancer associated myelopoiesis include: inflammatory cytokines such as IL-6, IL-1, and TNFα, chemokines such as CCL2, CCL3, and CCL4, growth factors G-CSF, GM-CSF, and others, and hormones and other mediators, such as estrogen, α-MSH, osteopontin, and angiotensin II. Such mediators may exert their effects on the hematopoietic system *via* both direct and indirect mechanisms. Cancer induced myelopoiesis and the production of tumor associated neutrophils and monocytes with immunosuppressive properties are promoted by the activation of unfolded protein response (UPR), including kinase PERK and transcription factors ATF4 and NRF2 in hematopoietic stem and progenitor cells (HSPCs). Cancer disease may also result in a long-term epigenetic reprogramming of HSPCs and the induction of aberrant trained immunity. This model integrates findings from multiple cancer types and experimental systems, and the indicated mechanisms may not be conserved across all cancers.

Recent decades saw rapid advances in our understanding of the immune mechanisms promoting cancer control and mediating tumor progression, and resulted in the development and adoption into clinical use of many novel immunotherapies (1, 2). In light of these advances, we must consider that most immune cells are short-lived and continuously replenished from hematopoietic stem and progenitor cells (HSPCs) (3, 4). Hematologic dysfunction is prevalent in cancer patients, and the elevated neutrophil-to-lymphocyte ratio is a common diagnostic and prognostic indicator (5–7). Importantly, in recent years hematopoietic dysfunction is increasingly recognized as more than a side-effect of cancer pathology, but as an active contributor to disease mechanisms and a target for therapeutic intervention (8, 9). Here we discuss hematopoietic dysfunction as one of the systemic features of cancer disease, covering data from patients and mouse models with solid tumors, but excluding from the discussion hematologic malignancies, tumor metastasis into the hematopoietic tissues, or side-effects of cancer therapy (10, 11). We include an in depth overview of the cancer associated HSPC phenotypes, myelopoiesis dysfunction, recent advances in the study of cancer associated aberrant erythropoiesis, and their contributions to immunosuppression (**Figures 1**, **2**). Cancer associated thymic dysfunction and any effects on the primary lymphocyte repertoire selection and cancer surveillance by the adaptive immune system are however beyond our scope and are reviewed elsewhere (2, 12, 13).

## Hematopoietic stem and progenitor cells in cancer

Hematopoietic stem and progenitor cells (HSPCs) are the precursors of all blood and most immune cell lineages, except for some pools of tissue-resident macrophages. HSPCs sense and respond to diverse stresses, danger signals, and inflammatory cytokines, and are recognized as active mediators of systemic immune and inflammatory response (14–16). Full understanding of antitumor immunity therefore requires in-depth knowledge of the effects of the tumor on the pathways guiding immune cell production and on the HSPC compartment.

Wu et al. demonstrated an increase in circulating HSPCs in patients across seven different cancer types, with a strong increase in granulocyte monocyte progenitors (GMPs), and lesser but significant increases in multipotent progenitors (MPPs) and hematopoietic stem cells (HSCs) (17). Importantly, the frequency of circulating GMPs correlated with advanced disease and predicted disease progression (17). HSPC expansion was also observed in the blood of rhabdomyosarcoma and breast cancer patients, and high circulating HSPCs at the time of diagnosis correlated with metastatic progression (18). Mobilized HSPCs are also detected within human tumors. In particular, the highly aggressive and therapy-resistant brain tumors glioblastomas harbor HSPCs with myeloid differentiation potential, immunomodulatory properties, and capacity to promote tumor cell proliferation in co-culture assays (19). Importantly, HSPC abundance in such tumors correlates with immunosuppressive phenotype, tumor grade, and poor prognosis for the patients (19). All this indicates that major dysregulation of hematopoiesis is prevalent in human cancer, and suggests its association with disease mechanisms.

Studies in mouse models provide further support for these conclusions. In the MMTV-PyMT transgenic mouse model of spontaneous breast cancer the accumulation of immunosuppressive myeloid cells within the tumor is preceded by an expansion of HSCs, MPPs, and GMPs in the bone marrow, driven by G-CSF and requiring G-CSFR expression on the progenitor cell populations (20). Major hematopoietic dysfunction is also observed in the MMTV-*neu* ^OTI/OTII^ and other mammary tumor models and in a mouse melanoma model induced *via* a subcutaneous injection of B16-F10 cells (21, 22). This includes an expansion of bone marrow MPP and GMP cells, anemia, thrombocytopenia, and induction of extramedullary myelopoiesis and erythropoiesis in the mouse spleen (21, 22). An independent study in mice subcutaneously inoculated with MC57 fibrosarcoma, B16-F10 melanoma, MC38 colon adenocarcinoma, or Lewis lung carcinoma (LLC) cells also demonstrated HSPC expansion and myeloid biased hematopoiesis, with increased HSPC proliferation and turnover (23). Similarly, mice with an orthotopic inoculation of E0771 breast carcinoma and M3-9-M rhabdomyosarcoma cells demonstrated HSC and MPP expansion in the bone marrow, their mobilization into the bloodstream, homing into tissues, and local differentiation into myeloid cells with immunosuppressive properties (18).

While the expansion of myeloid biased HSPCs is widely reported across the studies and cancer models discussed above, the systemic effect of cancer disease on lymphoid progenitors is less clear. For example, no consistent changes in the frequency of cells with the surface marker profile of common lymphoid progenitors (CLPs) are observed in the blood of patients across seven different cancer types (17), and no changes in the frequency of CLPs and lymphoid-biased Flt3^+^ MPPs are observed in the bone marrow of mice bearing MC57 fibrosarcoma tumors (23). Furthermore, patients with non-medullary thyroid carcinoma (TC) also showed no depletion of lymphoid progenitors and a trend toward expansion of B cell precursors based on single cell RNA-sequencing (scRNA-seq) of bone marrow mononuclear cell samples (24). However, cells with the CLP transcriptional signature are detected in human glioblastoma tumor samples albeit at low frequencies (19), suggesting that HSPC mobilization in cancer is not limited to myeloid-biased cells. Furthermore, in B16-F10 melanoma bearing mice a reduction in circulating lymphocytes and a depletion of bone marrow pre-B and immature B cells is observed, indicating dysregulation in lymphopoiesis (21). Similarly, in a murine cancer model induced with a subcutaneous injection of syngeneic EL4 thymoma cells a reduction in CLPs, NK precursors (CD122^+^NK1.1^-^CD3^-^), and NK cells and B cells is reported, indicating a dysfunction in lymphopoiesis (25).

Considering the widely reported expansion and mobilization of HSPCs across cancer models, it is also interesting to note that HSPCs express MHCII, co-stimulatory molecules (CD80, CD86) (26, 27), and immune checkpoint receptors (PD-L1) (28), and can act as bona fide antigen presenting cells (29). While this suggests that the mobilized HSPCs enriched in the blood, lymphoid organs, and the tumor tissues of cancer patients may similarly engage in modulation of immune response, given their low numbers it is difficult to assess if such activity is biologically significant. Overall, the immunomodulatory properties of HSPCs are likely exerted primarily *via* their differentiation into diverse immune cell lineages, rather than through direct engagement with immune cells.

## Aberrant myelopoiesis in cancer: roles and mechanisms

Myeloid biased hematopoiesis is a major feature of hematopoietic dysfunction in cancer. It produces heterogeneous populations of monocytes and neutrophils with immunosuppressive properties, and the abundance of such cells is a prognostic marker of poor outcomes in cancer patients (30, 31). The term myeloid-derived suppressor cells (MDSCs) has been widely used for such cells, despite their significant heterogeneity in surface markers, morphology, gene expression, and life-span (32). However, in recent years mass cytometry (CyTOF), scRNA-seq, lineage tracing, and other approaches have revolutionized our understanding of the heterogeneity of myeloid cell states and the plasticity of the developmental pathways driving their production in health and disease (33–38). These studies indicate MDSCs as aberrant myeloid cell states enriched in malignant disease, with some distinct transcriptional signatures and functional properties, but falling within the broader spectrum of myeloid cell heterogeneity (39, 40).

Most such cancer-associated monocytes and neutrophils are short-lived and continuously replenished from HSPC pools, and this highlights myelopoiesis dysfunction as key to the understanding of their origins (41). In this regard, recent studies saw the identification of specialized monocytic progenitor cells (MLPGs) that do not contribute to granulopoiesis under steady-state conditions but expand in the bone marrow and spleen of tumor-bearing mice and become an important precursor of tumor associate neutrophils (42). Other notable studies identified unipotent neutrophil progenitors in both mouse and human bone marrow (43–45), and demonstrated that such cells can promote tumor growth in humanized mouse models and are enriched in the blood of melanoma patients (44) and in the tumors of non-small cell lung carcinoma (NSCLC) patients (45). Here we review the role of aberrant myelopoiesis in cancer, covering the importance of extramedullary niches, the driving molecular mechanisms, and key evidence supporting its pathogenic role in the disease.

### Splenic extramedullary myelopoiesis in cancer

The spleen is recognized as an important site for the homing and proliferation of immunosuppressive myeloid cells in cancer (46), and also for the induction of tolerance to tumor antigens (47). Recent studies further demonstrate that the spleen is also the critical site of aberrant myelopoiesis that produces such pro-tumorigenic myeloid cells (48).

In the *Kras* ^LSL-G12D/+^ *p53* ^flox/flox^ (KP) mouse model, in which lung adenocarcinoma is induced through an intranasal administration of a Cre-expressing adenovirus, cancer progression is linked to the expansion of GMPs in the mouse spleen, leading to local differentiation of monocytes and neutrophils and their relocation into the tumor (49). Similarly, in an orthotopic mouse model of hepatocellular carcinoma (HCC), induced with a subcapsular intrahepatic injection of Hepa1-6 cells, a striking expansion of splenic HSPCs was observed (50). Such HSPCs retained their capacity for long-term self-renewal, but in contrast to bone marrow HSPCs, had myeloid restricted differentiation potential and gave rise to tumor associated neutrophils with potent immunosuppressive properties (50). These studies further demonstrated the accumulation of splenic HSPCs and myeloid progenitors in human cancer patients (49, 50), which correlated with myeloid cell expansion and in gastric cancer cohorts also with poor prognosis (50). Recently scRNA-seq of HSPCs (Lin^-^cKit^+^Sca1^+^) from the spleen and bone marrow in the orthotopic Hepa1-6 HCC mouse model demonstrated a strong expansion of myeloid biased cells in the spleen, resembling the MPP3 subset of bone marrow HSPCs, with the expression of many genes indicative of myeloid lineage priming (51). In contrast, in mice transgenic for a photoconvertible protein KikGR that allows to ‘timestamp’ myeloid cells with surgery and violet light exposure, subcutaneous inoculation of syngeneic LLC lung adenocarcinoma cells demonstrated that the bone marrow rather than spleen remained the major source of monocytes, including those infiltrating the tumors (52).

Interestingly, splenectomy in the KP lung adenocarcinoma mouse model impaired myeloid cell accumulation within the tumor and delayed tumor growth (49). Similarly, in the orthotopic Hepa1-6 HCC mouse model splenectomy effectively reduced the suppressive activity of neutrophils and increased the frequency of tumor-infiltrating IFNγ^+^ CD8 T cells. In this model splenectomy resulted in a mild but significant extension in mouse survival and had further synergistic effects when combined with anti-PD-L1 immunotherapy (50). Furthermore, in mice inoculated with EG7-OVA lymphoma cells *via* a subcutaneous injection splenectomy abolished the induction of T cell tolerance to model tumor antigens (47). However, the effects of splenectomy on cancer progression demonstrated variable (53–55) and in many cases deleterious (56, 57) outcomes across other mouse models and in cancer patients, indicating the need for more precise and refined interventions to counteract aberrant myelopoiesis in cancer.

Liver is also a major site of extramedullary hematopoiesis in many disorders. Accumulation of myeloid cells with immunosuppressive properties (CD11b^+^GR1^+^) within the liver is reported in mice inoculated subcutaneously with DA-3 and 4T1 mammary, B16-F10 melanoma, or LLC lung adenocarcinoma tumors (58). Furthermore, in DA-3 tumor bearing mice an enrichment of myeloid progenitors in the liver is demonstrated with colony forming assays (CFU-GEMM, CFU-GM), and is associated with an immunosuppressive reprogramming of Kupffer cells (58). Nevertheless, the role of the liver as a site of myelopoiesis and a source of immunosuppressive monocytes and neutrophils in cancer remains relatively poorly understood.

### Secreted mediators promoting cancer myelopoiesis

The growing understanding of the molecular signals that drive splenic HSPC recruitment and myelopoiesis in cancer may offer novel strategies for therapeutic intervention. Increased levels of growth factors and cytokines, such as GM-CSF, G-CSF, M-CSF, IL-6 and others, are commonly seen in cancer patients and mouse models, both systemically or in the tumor microenvironment (59–61). However, such mediators have pleiotropic effects that span hematopoiesis, immune cell development, the regulation of immune effector functions, as well as direct effects on the malignant cells within the tumor. Thus it is often challenging to selectively analyze the role of such secreted factors as drivers of the cancer associated myelopoiesis independently of their other functions, and to define the specific contribution of this activity to their pro-tumorigenic effects. Nevertheless, a number of important studies in this area are reviewed here.

Despite the largely redundant role of GM-CSF in steady-state myelopoiesis under homeostatic conditions (62, 63), it has more recently emerged as an important inflammatory mediator (64). The effects of GM-CSF in cancer onset and progression are widely studied, but complex and variable across cancer models and modes of administration (60, 61). GM-CSF has been shown to potentiate cancer associated myelopoiesis in several models (61). For example, in an orthotopic Hepa1-6 HCC mouse model splenic HSPCs express high levels of GM-CSF, and further GM-CSF supplementation in this model enhances, while its inhibition supresses the production of immunosuppressive neutrophils (CD11b^+^Ly6G^+^Ly6C^lo^) and other myeloid cells (CD11b^+^Gr-1^+^) (50). However, GM-CSF can have potent immunostimulatory and adjuvant properties, and early clinical trials administering cancer patients with irradiated GM-CSF-transduced tumor cells showed enhanced antitumor immunity and favourable outcomes, for example in metastatic melanoma (65, 66), non-small cell lung cancer (67), and prostate cancer (68). On the other hand, GM-CSF can also have direct effects on many cancer cells, promoting their proliferation and migration (60). The effects of GM-CSF on antitumor immunity may also encompass its role in the adaptive immune system, in particular in the induction and maintenance of Th17 T cells (69, 70). Overall, the multifunctional nature of GM-CSF makes it a highly challenging tool or target for therapeutic intervention.

M-CSF is a highly important mediator for the development and maintenance of monocytes and tissue resident macrophages (71). It is produced by many tumors (72), and its elevated serum levels or the high expression of its receptor CSF-1R on the tumor cells correlate with tumor grade and poor prognosis in cancer patients (73–75). Tumor M-CSF production also correlates with increased monocyte infiltration (76), can enhance the immunosuppressive properties of monocytic cells in some cancer models (77), although the expression of a membrane-bound form of M-CSF was also reported to promote tumor cell killing by macrophages (78). Therapeutic blockade of CSF-1R (79–82) or the inhibition of its signal transduction (83) have shown protective activity across different murine cancer models (84, 85), and are investigated in ongoing clinical trials (85). However, administration of M-CSF was also tested and promoted immune recovery from chemotherapy-induced immunosuppression in ovarian cancer patients (86), showed minimal adverse effects and some favorable outcomes in metastatic melanoma patients (87), and was not associated with increased risk of disease remission in hematologic cancers (88, 89). In summary, M-CSF is a well-established regulator of myeloid cell biology in health and disease; and given its pleiotropic functions it is often difficult to differentiate the role of M-CSF as a driver of myelopoiesis in cancer models from its other functions systemically and in the tumor microenvironment.

Other growth factors and cytokines are also implicated in hematopoietic dysfunction in cancer models. TNFα has been shown to promote HSPC activation and myelopoiesis in mice bearing subcutaneous MC57 fibrosarcoma tumors (23). TNFα is also essential for the accumulation of immunosuppressive myeloid cells (CD11b^+^GR1^+^) and for tumor growth in mice inoculated subcutaneously with FB61 fibrosarcoma or J558L plasmacytoma cells (90), and for disease progression in chemically-induced skin cancer models (91). A pharmacological blockade of either G-CSF or IL-1 was shown to normalize immune cell numbers across multiple tissues in eight murine tumor models (92). In the MMTV-PyMT transgenic breast cancer model HSPC expansion and the production of tumor associated neutrophils required G-CSFR expression (20). Moreover, tumor derived G-CSF in mice with orthotopic MMTV-PyMT mammary tumors was also implicated as the key factor repressing the development of the cDC1 subset of conventional dendritic cells, by downregulating the expression of IRF8 in cDC-precursors (93). Furthermore, IL-6 is implicated in promoting cDC1 apoptosis and depletion in the *Kras* ^LSL-G12D^ *p53* ^LSL-R172H/+^ Pdx1-CRE murine model of pancreatic ductal adenocarcinoma (PDAC) (94).

Chemokines and their receptors are also widely investigated for their roles in aberrant myelopoiesis in cancer. Thus, recent studies in the orthotopic Hepa1-6 HCC mouse model identify the CCL2/CCR2 axis as critical for HSPC recruitment to the spleen of tumor bearing mice, and establish its effective suppression by CCR2 antagonists (50). In other studies such CCR2 antagonists showed favourable activities beyond the repression of splenic HSPC recruitment and myelopoiesis, but also impaired myeloid cell survival, Treg homing, and tumor cell growth (95). High serum levels of CCL2 in renal cell carcinoma patients correlated with myeloid cell levels and were predictive of poor survival (47). Further studies demonstrated that HSPCs primed by tumor derived factors produce CCL3-4, which act *via* autocrine or paracrine mechanisms on CCR5 and CCR1 to promote the differentiation of tumor associated neutrophils with immunosuppressive properties (96). The silencing of CCR5 and CCR1 expression on myeloid cells, including the myeloid precursors, was sufficient to inhibit tumor progression across multiple mouse models (96).

A number of secreted mediators with primary functions beyond immune regulation are also implicated as drivers of aberrant myelopoiesis in cancer. Thus in mice inoculated subcutaneously with CT26 colon carcinoma, tumor-derived osteopontin promoted extramedullary myelopoiesis, while its antibody-mediated blockade *in vivo* effectively inhibited tumor growth (97). Furthermore, in the KP lung adenocarcinoma model tumor-derived angiotensin II promoted HSPC expansion, splenic accumulation, and myeloid differentiation, while treatment with an inhibitor of angiotensin converting enzyme suppressed the aberrant myelopoiesis and significantly delayed tumor-induced mortality (98). Estrogen also promotes cancer progression in mice bearing intraperitoneal syngeneic ID8-*Defb29/Vegf-a* ovarian cancer tumors and in several heterotopic murine cancer models, and this effect can be independent of estrogen receptor expression by the tumor, but require its expression on the hematopoietic and immune cells. This study indicated that estrogen can act on myeloid progenitors in the bone marrow of tumor bearing mice to promote aberrant myelopoiesis and inhibit antitumor immunity (99). Similarly, the production of α-melanocyte stimulating hormone (α-MSH) by the pituitary gland was shown to promote HSPC expansion and cancer myelopoiesis, and to repress antitumor immunity across several murine cancer models, with α-MSH acting on the MC5R receptor on HSPCs (100). Increased α-MSH serum levels were also observed in cancer patients and correlated with myeloid cell expansion, suggesting that similar neuroendocrine mechanisms may promote cancer myelopoiesis in human (100).

### Signaling pathways regulating cancer myelopoiesis

Recent studies demonstrated the key role of unfolded protein response (UPR) and its mediator kinase PERK in the production and immunosuppressive activity of tumor associated neutrophils and monocytes (101). Various experiments in this study utilized mice inoculated subcutaneously with LLC, B16-F10, or EG7 tumor cells, intraperitoneally with ovarian carcinoma cells, as well as the KP mouse model (*Kras* ^LSL-G12D/+^ *p53* ^flox/flox^) receiving an intramuscular delivery of a Cre-expressing adenovirus for sarcoma induction (101). Both a pharmacological inhibition of PERK and its selective deletion within the myeloid cell lineage resulted in enhanced antitumor immunity and slowed disease progression across multiple cancer models, and this protective activity was associated with the repression of NRF2 and upregulation of STING signaling in myeloid cells (101).

Further studies in an orthotopic Hepa1-6 hepatoma mouse model using a micro-osmotic pump for targeted PERK inhibitor delivery demonstrated that the delivery of the inhibitor to the spleen rather than the tumor was most effective at limiting cancer associated myelopoiesis, and resulted in enhanced tumor infiltration by IFNγ^+^ CD8 T cells and delayed disease progression (51). Mouse HSPCs co-cultured with splenic stromal cells from tumor bearing mice or human cord blood HSPCs cultured with IL-6 and other cytokines demonstrated activation of PERK and its downstream signaling mediators, and gave rise to neutrophils with immunosuppressive properties in a PERK dependent manner (51). Furthermore, PERK activation was observed in splenic HSPCs of patients with hepatocellular carcinoma and gastric cancer, and correlated with myeloid cell abundance. Overall, this indicates PERK as a promising drug target to prevent cancer immune evasion (51). This study also supports the specialized role of the splenic environment and the aberrant signaling events in splenic HSPCs as drivers of cancer associated myelopoiesis in hepatocellular carcinoma (51), although it remains to be explored to what extent the same mechanisms apply in other cancer models.

Pattern recognition receptors (PRRs) comprise multiple families of cell-surface, endocytic, and intracellular proteins responsible for the sensing of microbial compounds and endogenous danger signals (102), leading to the activation of innate immune and inflammatory responses (103). HSPCs express diverse PRRs, and cell-intrinsic PRR stimulation on HSPCs contributes to the induction of emergency myelopoiesis in response to infections (15, 104). In the context of cancer, PRR functions are widely investigated in tumor cells and in tumor infiltrating leukocytes (TILs) (103, 105, 106), however whether cell-intrinsic stimulation of certain PRRs on HSPCs contributes to the induction of cancer-associated emergency myelopoiesis and immunosuppression remains poorly understood.

Several other novel pathways were recently identified as important for cancer associated myelopoiesis and immunosuppression. In the MMTV-PyMT mammary carcinoma mouse model retinoic-acid-related orphan receptor RORC1 was found to be essential for the differentiation of tumor associated neutrophils and monocytes, and its ablation in the hematopoietic compartment inhibited tumor growth and metastasis (107). Recent studies further demonstrated the essential role of the fatty acid transport protein 2 (FATP2) in tumor associated neutrophils across multiple murine cancer models, including mice inoculated subcutaneously with EL4 (lymphoma), LLC (Lewis lung carcinoma), CT26 (colon carcinoma), and TC-1 (lung epithelial cancer) cells (108). FATP2 was shown to mediate arachidonic acid uptake and facilitate prostaglandin E2 production, although its roles in myelopoiesis were not fully explored. FATP2 pharmacological inhibition impaired the suppressive activities of tumor associated neutrophils and delayed tumor progression, both alone and in synergy with other forms of immunotherapy (108), suggesting novel avenues for cancer treatment.

### Epigenetic reprogramming and trained immunity in cancer myelopoiesis

Induction of emergency myelopoiesis in the context of infectious diseases and some chronic inflammatory disorders is characterized by long-term epigenetic reprogramming of HSPCs that can result in altered immune responses to subsequent challenges (109–112), and is collectively described as trained immunity (113). Early studies in MMTV-*neu* ^OTI/OTII^ and other mammary tumor models demonstrated alterations in the overall levels of histone modifications in HSPCs, including H3K27me3 and H3K4me3 (22). Such changes were reproduced with bone marrow exposure to tumor conditioned media, and correlated with altered expression of genes encoding essential epigenetic and transcriptional regulators, like *Ezh2* and *Hoxa9* (22). Overall, this supports epigenetic reprogramming of HSPCs as a possible contributing mechanism for hematopoietic and immune dysfunction in cancer.

Recent studies provide further definitive demonstration that trained immunity can be induced in cancer models, with both protective and deleterious outcomes. Thus, pre-treatment with fungal β-glucan, which is a prototypic inducer of trained immunity, resulted in impaired growth of B16-F10 melanoma and LLC lung adenocarcinoma tumors following subcutaneous inoculation into mouse models (114). These effects were independent of the adaptive immune system, required type-I interferon signaling, and involved large-scale transcriptional and epigenetic reprogramming of bone marrow granulopoiesis (114). The protective trained immunity was transferred to recipient mice with adoptive transfers of the trained neutrophils, and with bone marrow transplantation from the β-glucan treated donors, persisting for at least 6 weeks post-transplantation (114). In a related study, β-glucan pre-treatment was also shown to promote hematopoietic recovery following myeloablative treatment, further supporting its potential favourable effects in the context of cancer therapy (110). In contrast, studies addressing the mechanisms of accelerated breast cancer progression following myocardial infarction (MI), using either MMTV-PyMT transgenic mice or mice inoculated orthotopically with a mammary cancer cell line E0771, demonstrated an expansion of Ly6C^hi^ monocytes in mouse bone marrow, blood, and tumor tissues. Such Ly6C^hi^ monocytes had altered chromatin accessibility (ATAC-seq) at the genes engaged in ER-stress response, UPR, and inflammation (115), which are also implicated in the induction of aberrant myelopoiesis in other cancer models (51, 101). Depletion of the Ly6C^hi^ cells abrogated the MI-induced increase in breast cancer progression, supporting their pathogenic activity (115). While the epigenetic analyses in this study did not encompass HSPCs, mouse-to-mouse bone marrow transplantation showed enhanced tumor growth in mice grafted with bone marrow from MI-donors, supporting long-term HSPC reprogramming and the induction of deleterious tumor-promoting trained immunity in this model (115).

Other studies however argue against stable reprogramming of immune function in cancer, and instead demonstrate its highly dynamic regulation. For example, recent immune profiling across multiple tissues in eight murine tumor models demonstrated reversion of the major disease associated changes in immune cell states with tumor resection or blockade of G-CSF or IL-1 signaling (92). However, such dynamic regulation does not rule out that cancer associated epigenetic changes may persist in HSPCs and affect immune responses to subsequent challenges. Systematic comparative analyses of HSPC reprogramming and immune dysfunction across cancer models and other established models of trained immunity in infectious and chronic diseases may provide further insights in this area and suggest new avenues for therapeutic intervention.

### Cancer regulation of the hematopoietic niche induces aberrant myelopoiesis

HSPC activities are tightly regulated by their niche (116); and the aberrant myelopoiesis and hematopoietic dysfunction in cancer may therefore also involve complex effects of the tumor on the cellular components of the hematopoietic niche. For example, in lung adenocarcinoma models in KP (*Kras* ^LSL-G12D/+^ *p53* ^flox/flox^) mice or mice injected intravenously with LLC or KP1.9 tumor cells the induction of osteocalcin-expressing osteoblasts promotes the development of SiglecF^high^ neutrophils, with distinct transcriptional profiles and cancer-promoting properties (117). Increased trabecular bone density is also reported in lung adenocarcinoma patients, and an enrichment of SiglecF^high^ neutrophil transcriptional signatures in patient tumors is associated with deleterious outcomes, indicating that similar mechanisms are relevant in human (117). Furthermore, the protective effect of bisphosphonates, such as zoledronic acid, against breast cancer onset and recurrence are reported to be mediated *via* a reduction in osteoclast numbers and activity in the hematopoietic bone marrow niche, with downstream effects on HSPCs and myelopoiesis (118). Furthermore, melanoma derived exosomes were reported to reprogram bone marrow vascular endothelial progenitors to promote tumor metastasis (119, 120). Studies in the B16-F10 melanoma bearing mice also showed that fibroblastic reticular cells undergo proliferation, structural remodelling, and large-scale transcriptional reprogramming in the tumor draining relative to control lymph nodes (121), and this is characterized by downregulation of cytokine and chemokine gene expression and may contribute to cancer immune evasion (121).

### Cancer myelopoiesis and the ontogeny of tumor associated macrophages

Tumor associated macrophages (TAMs) are heterogeneous in their ontogeny, and include both monocyte derived macrophages (MoD-TAMs) and the expanded populations of tissue resident macrophages (Res-TAMs) (122, 123). In many tissues such resident macrophages originate from embryonic and fetal hematopoietic progenitors and self-renew locally and independently of bone marrow hematopoiesis (124, 125). The abundance of these developmentally distinct macrophage subsets differs between tissues, with the age of the host, as well as across cancer models. It is increasingly studied in murine models using lineage tracing, parabiosis, and chimera systems (122, 123), and also by analyzing for TAM depletion in mice with the disruption of the CCL2/CCR2 chemokine axis that plays a major role in monocyte recruitment into tissues (126). Here we review the evidence for the importance of TAMs of both monocyte derived (MoD-TAM) and resident macrophage (Res-TAM) origins in different murine tumor models.

In the KPC mouse model of spontaneous pancreatic ductal adenocarcinoma (*Kras* ^LSL-G12D^ *p53* ^flox/+^ p48-CRE) distinct TAMs originated from monocytes and from resident macrophages of embryonic ontogeny, and of these MoD-TAMs were reported to have stronger capacity for antigen presentation, while Res-TAMs exhibited a pro-fibrotic transcriptional signature (127). Similarly, in a murine lung cancer model, induced with an intravenous injection of TC-1 lung epithelial cells transformed with the c-Ha-Ras and HPV16 E6 and E7 oncogenes, distinct Res-TAMs and MoD-TAMs were observed with different abundance based on the location of the tumor nodules and distance from the vasculature (128). Both TAM subsets were sensitive to cyclophosphamide chemotherapy, however only the MoD-TAMs rapidly recovered in numbers and promoted phagocytic clearance of cancer cells after the treatment (128). In contrast, in the *Apc* ^Min/+^ murine model of spontaneous or DSS-induced colon adenoma CCR2-independent Res-TAMs were shown to self-renew and expand within the tumors independently of bone marrow hematopoiesis (129). Furthermore, in a mouse model of metastatic ovarian cancer induced with an intraperitoneal injection of ID8 tumor cells, Res-TAMs in the omentum with embryonic ontogeny and a CD163^+^ Tim4^+^ cell surface marker profile were shown to promote metastatic disease progression (130).

TAM ontogeny is also widely studied in the MMTV-PyMT mouse model that develops spontaneous mammary tumors. MoD-TAMs were shown to be an important immunosuppressive macrophage population particularly within advanced tumors in this model, and their depletion could enhance CD8 T cell infiltration and suppress tumor growth (131). Further studies also characterized the heterogeneity among Res-TAMs in this model in gene expression, tissue localization, and functional properties. Such heterogeneous Res-TAMs were suggested to arise from the stromal and ductal populations of resident mammary macrophages, and had distinct capacity for tumor cell phagocytosis and CD8 T cell activation (132).

TAM ontogeny is also extensively studied across glioma models. Lineage tracing studies in mouse glioma induced through an orthotopic intracranial tumor cell injection demonstrated the dominant role of monocytes as TAM precursors (133–135). Such studies defined the distinct transcriptional and epigenetic signatures of MoD-TAMs and microglia derived Res-TAMs, and *Itga4*/Cd49d as a marker of MoD-TAMs in glioma in both mouse and human (133). Studies in xenograft models demonstrated that the microglia derived Res-TAMs are the dominant effector cells that mediate glioma cell phagocytosis in response to CD47-blockade therapy, to extend mouse survival (136). Furthermore, transcriptional signature of MoD-TAMs rather than microglia-derived TAMs correlated with poor prognosis in human glioma (137).

It is interesting to note that there is no strong correlation between TAM ontogeny and their pro- versus anti-carcinogenic functions across the different models and studies discussed above (122, 123). We can therefore conclude that ontogeny is only one factor accounting for TAM heterogeneity, and local education within the tumor microenvironment plays a major role in defining TAM functional properties. However, an in depth discussion of TAM education within the tumor and the epigenetic, metabolic and other mechanisms that reprogram both infiltrating monocytes and resident macrophages with cancer onset and progression are beyond the scope of the current review, and have been reviewed elsewhere (138). It is also important to note that TAM ontogeny is highly challenging to study in human. With the growing use of transcriptional data for TAM classification, commonalities are starting to emerge between mouse and human (37, 38, 137), however it remains to be established whether the developmental origins of human TAMs can be inferred based on shared transcriptional signatures with mouse studies.

## Erythropoiesis dysfunction contributes to cancer immunosuppression

While anemia has been recognized as a common feature of advanced cancer for many decades (139–141), recent studies shed new mechanistic insights into the cellular and molecular dysregulation of erythropoiesis in cancer, and into its unexpected roles as a mediator of cancer progression. Thus, in mouse models inoculated subcutaneously with Lewis lung carcinoma (LLC) or B16-F10 melanoma, disease progression was linked to an accumulation of erythroid progenitor cells (EPCs) in the mouse spleen (142). Unlike splenic EPCs observed in extramedullary erythropoiesis of severe anemia, the splenic EPCs of tumor bearing mice had strong immunosuppressive properties, inhibiting proliferation and cytotoxic activity of CD8 T cells *ex vivo*, and accelerating melanoma progression *in vivo*. Importantly, such aberrant EPCs capable of repressing CD8 T cell proliferation were also observed in the blood of cancer patients and their abundance correlated with both anemia and immunosuppression (142).

Further work by the same team demonstrated a major restructuring of the hierarchical organization of erythropoiesis in patients with advanced cervical carcinoma and in mouse models, including the MMTV-PyMT transgenic model of spontaneous breast cancer and mice inoculated subcutaneously with LLC lung carcinoma, B16-F10 melanoma, or MC38 colon adenocarcinoma (143). Both patients and mouse models with advanced cancer harboured myeloid cells that co-expressed markers and transcriptional signatures of the erythroid cell lineage (143). Furthermore, EPCs in these models, but not in the corresponding healthy controls, had a significant potential for myeloid differentiation, and this was further enhanced with GM-CSF exposure or transplantation into tumor bearing mice. The resulting erythroid differentiated myeloid cells (EDMCs) had potent immunosuppressive activities even in comparison to conventional myeloid cells (CD11b^+^Gr1^+^TER119^-^CD71^-^) from the same model, suppressing CD8 T cell proliferation and IFNγ production in *ex vivo* assays. Furthermore, when administered to mice EDMCs promoted melanoma metastasis and resistance to immune checkpoint inhibitor (ICI) therapy. Importantly, EDMC transcriptional signatures in human cancers also correlated with CD8 T cell exhaustion, other markers of cancer immune evasion, and with an impaired response to ICI therapy (143).

A related population of splenic cancer promoting erythroblast-like cells, known as Ter-cells, was characterized in parallel studies in a mouse orthotopic hepatocellular carcinoma (HCC) model, in mice inoculated subcutaneously with EG7 T lymphoma or B16-F10 melanoma cells, as well as in a diethylnitrosamine-induced primary HCC (144) and xenograft HCC mouse models (144). These cells displayed Ter119^+^CD45^-^CD71^+^CD41^+^CD44^+^ cell surface markers and a transcriptional profile resembling megakaryocyte-erythroid progenitors (MEPs), with the co-expression of genes of erythroid and megakaryocyte, but not myeloid lineages (145). The cells were also characterized by their high expression of the neurotrophic secreted mediator artemin. In the mouse models, Ter-cell derived artemin promoted cancer progression, while artemin knockout, pharmacological inactivation, or tumor-restricted knockdown of its receptor GFRα3 delayed cancer progression (145). Importantly, increase in serum artemin levels and elevated tumor expression of GFRα3 were also observed in human HCC patients and correlated with poor prognosis (145). The exact developmental and functional relationships of EPCs, EDMCs, and Ter-cells with each other (142, 143, 145), as well as with the rare extramedullary and immunomodulatory subsets of erythroid cells observed under healthy homeostatic hematopoiesis in human and mouse (146–149) remain to be addressed in future work.

The secreted mediators and signaling pathways driving such cancer associated erythropoiesis dysfunction are not well known, however a role for the platelet-derived growth factor family member PDGF-BB in promoting extramedullary erythropoiesis was previously proposed (150). In this study tumor specific overexpression of PDGF-BB in the T241 fibrosarcoma and LLC lung carcinoma cells subcutaneously inoculated into mouse models was shown to induce erythropoietin production by stromal cells, stimulating extramedullary erythropoiesis in both spleen and liver, and promoting angiogenesis and tumor growth (150).

## Development of other immune and hematopoietic cell lineages in cancer

Comparatively less is known about the effects of cancer associated hematopoietic dysfunction on the development of other blood and immune cell lineages, but some recent highly relevant studies, as well as discussion points are included here. Thymic dysfunction, primary lymphocyte repertoire selection, and cancer immunosurveillance by the adaptive immune system are beyond our scope and are reviewed elsewhere (2, 12, 13).

### Dendritic cells

Dendritic cells (DCs) are the major professional antigen presenting cells, critical for the activation of naïve T cells and the induction of adaptive immunity. DCs therefore play a key role in antitumor immune response and in the response to cancer immunotherapy (151, 152). Tumor microenvironment can however impair DC infiltration and activation, and induce immunosuppressive DC states (153). At steady state DCs can be broadly classified into the conventional (cDC) and plasmacytoid (pDC) subsets, but under inflammatory conditions also include a monocyte derived DC (MoDC) subset. Many recent scRNA-seq studies provide us with a deep understanding of the heterogeneity of DC states in human cancer, and a meta-analysis of such data across human lung, breast, liver, colorectal, and ovarian tumors has defined cDC1, cDC2, DC3, pDC, and cDC2/MoDC cell subsets and their transcriptional signatures (154). Of these, cDC1 cells in particular are considered critical for the priming of CD8 T cells and for effective induction of antitumor immunity (155). Less is however known about how tumors affect systemic DC development.

cDCs originate from the common myeloid (CMP) and the downstream common DC (CDP) progenitors (156). They can commit to the cDC1 or cDC2 fate in the bone marrow (157), however exit bone marrow as pre-cDCs that retain proliferative capacity and complete their maturation within tissues. cDCs have a limited lifespan (10-30 days in mice) and are therefore maintained through ongoing HSPC differentiation (158, 159). Dysregulation in DC development has been reported in the context of systemic inflammation, including infections and sepsis (160, 161). This and the major dysfunction in myelopoiesis across the many cancer models suggests that DC development in cancer may be similarly affected. Indeed, a depletion of cDC precursors, including CDPs and pre-DCs, was observed in the bone marrow of patients with breast cancer and pancreatic ductal adenocarcinoma (PDAC), and correlated with a systemic reduction in cDC1 cells and a poor response to therapy (93). A depletion of cDC precursors and cDC1 cells in this study was also observed in mouse models, including transgenic models of breast cancer (MMTV-PyMT FvB/N) and PDAC (*Kras* ^LSL-G12D^ *p53* ^flox/+^ p48-CRE), and in mice with an orthotopic engraftment of syngeneic breast cancer or PDAC cells. Myeloid progenitors in mice with such MMTV-PyMT tumors were impaired in their capacity for cDC1 differentiation, and primed for granulocyte production. The defect in cDC1 development was mediated by tumor-derived G-CSF and correlated with impaired CD8 T cell mediated antitumor immunity (93). Thus G-CSF blockade with a neutralizing antibody could restore cDC1 numbers, and synergize with Flt3L and immunotherapy to promote antitumor immunity in this mouse model (93). Mechanistically, G-CSF was shown to downregulate the expression of the IRF8 transcription factor in DC precursors (93); and the low IRF8 expression in pre-DCs in cancer patients correlated with poor prognosis, indicating the relevance of such mechanisms in human (93).

In a related study in the KPC (*Kras* ^LSL-G12D^ *p53* ^LSL-R172H/+^ Pdx1-CRE) mouse model of PDAC cancer a systemic decline in cDC1 cells with disease progression was also reported, however here it was not associated with a significant depletion of cDC precursors or abnormalities in cDC1 development in the bone marrow. Instead cDC1 loss in this study was attributed to increased cell apoptosis, and could be rescued with a blockade of IL-6 (94). Nevertheless, treatment with Flt3L to stimulate DC development had a favourable effect in a related KPC (*Kras* ^LSL-G12D^ *p53* ^flox/+^ p48-CRE) mouse model when applied at early stages of tumorigenesis, and synergized with anti-CD40, STING-agonist, and radiotherapy in the treatment of advanced tumors, promoting the infiltration of cDCs and CD8 T cells and antitumor immunity (162). Similarly, in mice bearing subcutaneous B16 melanomas and in the transgenic *Tyr : CreER Braf* ^CA^ *Pten* ^loxP^ *Ctnnb1* ^lox-ex3^ mouse model where melanoma is induced with a cutaneous application of 4-hydroxytamoxifen, Flt3L treatment expanded pre-DCs and DCs both in the bone marrow and systemically, and resulted in enhanced antitumor immunity and delayed tumor growth in synergy with anti-PD-L1 and poly(I:C) immunotherapy (163). All this indicates stimulation of DC development as an approach for cancer therapy.

Recent studies have applied cutting-edge cellular barcoding and fate mapping approaches in mouse models to study the so-called “emergency DCpoiesis” induced either with Flt3L-treatment (164) or influenza A viral infection (165). Thus HSPC barcoding demonstrated a clonal expansion of cDC1-primed HSPCs and an enhanced cDC1 output with Flt3L treatment, and no associated defects in the production of other blood and immune cell lineages (164). Furthermore, *in situ* cDC fate mapping using *Clec9a* ^Cre^ *Rosa26* ^Confetti^ mice demonstrated an increased turnover of cDC clones and influx of pre-cDCs from the bone marrow with influenza A infection (165). Applications of similar methods in murine cancer models could be highly revealing to further address the effects of cancer disease and therapy on DC development.

### Platelets

Platelets are widely recognized as important mediators of immune and inflammatory responses (166, 167). In the context of cancer, platelets engage with both tumor cells and immune cells through the formation of tumor cell induced platelet aggregates (TCIPA), platelet-derived microvesicles (PMVs) (168), and secreted mediators. Overall, such interactions promote cancer progression and metastasis (169, 170). Platelet production holds a unique place in the hierarchical organization of hematopoiesis, due to their steady-state output from megakaryocyte-biased HSCs, independently of bi-potent megakaryocyte-erythroid progenitors (MEP) or other hematopoietic lineages (171, 172). Furthermore, under homeostatic conditions efficient platelet production can take place outside of the bone marrow, as for example in healthy lungs (173, 174). How cancer associated hematopoietic dysfunction affects platelet differentiation pathways, and the impact of such mechanisms on antitumor immunity and cancer progression remain to be addressed in future work.

## Cross-talk of immunity, hematopoiesis, and other physiological systems in cancer

Greater understanding of the systemic interactions between tumors, immunity, hematopoiesis, and other physiological systems promises further advances in the understanding of cancer disease mechanisms. For example, microbiota, obesity, and cardiovascular disease can all have complex effects on antitumor immunity (175–177) and also on hematopoiesis (178–180) across various studies. The link between increased risk of breast cancer progression and obesity or myocardial infarction is known to be mediated by pro-tumorigenic myeloid cell populations, which implicates myelopoiesis dysfunction as an important component of such mechanisms (115, 181, 182). Further dissection of the systemic crosstalk between antitumor immunity and hematopoiesis in cancer models may provide deeper understanding the mechanisms governing disease outcomes and suggest new strategies for therapeutic intervention.

Hematopoietic dysfunction in cancer patients is commonly exacerbated by the damage to the hematopoietic system from chemotherapy and radiotherapy regimens (10). These have complex effects on antitumor immunity, inducing immunogenic cancer cell death to prime the immune response against tumor antigens (183–185), while also disrupting the production of both immunogenic and immunosuppressive leukocyte subsets (186, 187). Such regimens also increase the incidence of clonal hematopoiesis among cancer patients and predispose to therapy-related myeloid neoplasms (11, 188, 189). The growing understanding of the interplay between the physiology of the hematopoietic process, the immune system, and the tumor promises the development of optimized therapeutic regimens to maximize cytotoxic activity against the tumor, while also preserving hematopoietic function and boosting antitumor immunity.

## Author contributions

All authors contributed to the article and approved the submitted version.
